# Cytokine profile levels and their relationship with parasitemia and cardiomyopathy in people with Chagas disease in Spain. A prospective observational study

**DOI:** 10.1007/s00436-023-08042-8

**Published:** 2023-12-22

**Authors:** Jose-Manuel Ramos-Rincon, Diego Torrús-Tendero, Hilarion García-Morante, Adelina Gimeno-Gascón, Francisco Marco, Concepción Gil-Anguita, Philip Wikman-Jorgensen, Ana Lucas-Dato, Juan-Carlos Rodriguez-Diaz, Concepción Amador, Jara Llenas-García

**Affiliations:** 1 Internal Medicine Department, Dr. Balmis General University Hospital, Alicante Institute for Health and Biomedical Research (ISABIAL), Pintor Baeza 12, 03010 Alicante, Spain; 2 Clinical Medicine Department, Miguel Hernández University of Elche, Alicante, Spain; 3 Reference Unit of Imported Diseases and International Health & Infectious Diseases Unit, Dr. Balmis General University Hospital, Alicante Institute for Health and Biomedical Research (ISABIAL), Alicante, Spain; 4 Parasitology Area, Miguel Hernández University of Elche, Alicante, Spain; 5 Internal Medicine Department, Vega Baja Hospital, Orihuela, Alicante, Spain; 6 Foundation for the Promotion of Health and Biomedical Research of the Valencia Region (FISABIO), Valencia, Spain; 7 Clinical Unit of Infectious Diseases, Microbiology, and Parasitology, Virgen del Rocío University Hospital, Seville, Spain; 8 Clinical and Molecular Microbiology Research Group, Institute of Biomedicine of Seville (IBiS), Seville, Spain; 9 Immunology Department, Dr. Balmis General University Hospital, Alicante Institute for Health and Biomedical Research (ISABIAL), Alicante, Spain; 10 Internal Medicine Department, Marina Baixa Hospital, Villajoyosa, Alicante, Spain; 11 Internal Medicine Department, General University Hospital of Elda, Alicante, Spain; 12 Microbiology Department, Dr. Balmis General University Hospital, Alicante Institute for Health and Biomedical Research (ISABIAL), Alicante, Spain; 13 Microbiology Department, Miguel Hernández University of Elche, Elche, Spain

**Keywords:** Chagas disease, *Trypanosoma cruzi*, Cytokines, Immunomodulation, Parasitemia, Chagas cardiomyopathy

## Abstract

Immunoregulatory networks may have a role in controlling parasitemia in the chronic phase of human Chagas disease. The aim was to describe the serum cytokine profile of *Trypanosoma cruzi* in chronically infected patients and to evaluate its relationship with parasitemia and Chagas cardiomyopathy.

This prospective observational study included adult patients with chronic Chagas disease. Demographic and clinical data were collected, and peripheral blood samples were used to perform *T. cruzi* real-time polymerase chain reaction (RT-PCR) and determine the serum cytokine profile.

Fifty-eight patients were included; 17 (29.3%) had positive RT-PCR results. This group had a higher median concentration of TNF-α (*p* = 0.003), IL-6 (*p* = 0.021), IL-4 (*p* = 0.031), IL-1β (*p* = 0.036), and IL-17A (*p* = 0.043) than those with a negative RT-PCR. Patients with cardiac involvement had a higher median concentration of IL-5 (*p* = 0.016) than those without*.*

These results reinforce the key role that cytokines play in Chagas disease patients with parasitemia and cardiac involvement.

## Introduction

Chagas disease is caused by the parasite *Trypanosoma cruzi*. According to the World Health Organization (WHO), there are an estimated 6–7 million people infected with this chronic disease in the endemic regions of continental Latin America (WHO 2015). Transmission is mainly vector-borne, although infection via blood transfusion, organ transplantation, and mother-to-child transmission can also occur (Pérez-Molina and Molina 2018). In recent years, migration and international travel by chronically infected and asymptomatic people have led to the globalization of the disease (Schmunis and Yadon 2010), which has now spread worldwide (Llenas-García et al. 2021).

After an acute infectious phase with initial parasitemia that can last four to 8 weeks, Chagas takes an asymptomatic, indeterminate form that can last 10 to 30 years (Pérez-Molina and Molina 2018). In the long term, the disease can affect different organs, especially through cardiomyopathy or megaviscera (mega-esophagus or/and megacolon) (Pérez-Molina and Molina 2018). High parasitemia characterizes both the acute phase and reactivations due to immunosuppression, while parasitemia is low and intermittent in the chronic phase. Molecular assays such as real-time polymerase chain reaction (RT-PCR) can detect the parasite in peripheral blood and are currently used to diagnose acute phase and early congenital Chagas disease and to check for treatment failure (Pérez-Molina and Molina 2018).

Children with acute Chagas disease have shown significantly increased levels of soluble IL-2 receptor (sIL-2R), sCD8 tumor necrosis factor (TNF)-α, IL-6, and sCD4. By contrast, levels of sIL-2R and sCD8 decrease after therapy, suggesting that those two cytokines could be a useful tool for following children with Chagas disease (Moretti et al. 2002).

Immunoregulatory networks may have a role in controlling parasitemia during the indeterminate or chronic phase of Chagas disease; however, there is still little information in this regard. In some studies, patients with positive RT-PCR for *T. cruzi* presented an elevated median concentration of the anti-inflammatory cytokine interleukin (IL)-10 (Salvador et al. 2020). In the indeterminate form, anti-inflammatory cytokines (IL-10, IL-17) may help neutralize the action of pro-inflammatory cytokines (IFN γ, TNF), and consequently may lead to reduced tissue damage and the absence of symptoms (Cristovão-Silva et al. 2021). On the other hand, there is some information about cytokines expression in plasma or serum and its association with Chagas cardiomyopathy. In the largest study, with 176 patients, IL-10 expression was higher in the indeterminate form, while inflammatory cytokine expression (such as IFN-γ, TNF-α, IL-6 and IL-1β) proved to be the highest in the cardiac form (Sousa et al. 2014; Vasconcelos et al. 2015). Another study showed that lower serum IL-10 levels could be associated with a severely impaired cardiac function (Vasconcelos et al. 2015). Moreover, in a substudy of the BENEFIT TRIAL with 109 patients, those with Chagas cardiomyopathy showed higher pro-inflammatory and lower anti-inflammatory cytokines; the results suggest the occurrence of specific immune responses, probably associated with different *T. cruzi* discrete typing units (Poveda et al. 2014). In one study in Brazil, people with Chagas cardiomyopathy showed a predominant Th1 immune response and increased levels of inflammatory cytokines, including IFN-γ (Gomes et al. 2003). Another study in Brazil with 32 patients showed that predominance of IFN-γ production over interleukin-10 (IL-10) production in antigen-specific cultures was associated with cardiac involvement (Llaguno et al. 2019).

This study aims to describe the serum cytokine profile of patients with a chronic *T. cruzi* infection and to evaluate its relationship with parasitemia and Chagas cardiomyopathy in a non-endemic setting.

## Materials and methods

### Study design and setting

A multicenter prospective observational study was conducted from July 2015 to December 2019 at four hospitals in Alicante (Spain): Alicante General University Hospital, Vega Baja Hospital, Marina Baixa Hospital, and Elche General University Hospital.

### Participants

Adult patients (≥18 years old) diagnosed with Chagas disease were eligible. Inclusion criteria were the following: agreeing to participate, signing informed consent, and having a *T. cruzi* RT-PCR result and a frozen serum sample to perform pre-treatment cytokine profile analysis. Exclusion criteria were the following: patients previously treated for Chagas disease, no baseline sample for pre-treatment cytokine analysis, and lack of RT-PCR for *T. cruzi*.

A confirmed diagnosis of Chagas disease was defined as two different positive serological tests, in line with World Health Organization (WHO) recommendations: Alicante University General Hospital used chemiluminescence immunoassay and ELISA, while the other three participating centers used chemiluminescence immunoassay and immunochromatography. Patients were treated for at least 30 days and underwent post-treatment follow-up analysis of the cytokine profile in serum.

### Variables

A standardized form was used to record the following variables: age, sex, country and area of residence in the country of origin, municipality of residence in Spain, year of arrival in Spain, comorbidities (hypertension, obesity, chronic heart disease, chronic lung disease, chronic liver disease, and type 2 diabetes mellitus), knowledge of Chagas disease, contact with a diagnosed family member, characteristics of the dwelling in the country of origin (rural/urban area, residence in adobe houses with a shanty roof), history of blood transfusion, most probable mechanism of infection, country of birth, and date and reason for attending the outpatient clinic.

A standardized form was used to record the following clinical history variables: any cardiological (palpitations, chest pain, dyspnea, syncope, edema, and stroke) and gastrointestinal symptoms (constipation, dysphagia, and heartburn). Both epidemiological and clinical data were collected prospectively.

All patients were offered treatment with benznidazole (300 mg/day b.i.d. or t.i.d.) and the duration of treatment, its completion, the cause of deprescription, and adverse effects were assessed. Patients intolerant to benznidazole were treated with nifurtimox. Treatment with benznidazole was in gradually increasing doses (treatment was started with 50 mg per day and then increased by 50 mg every day until 300 mg/day), as Navarrete et al. (2016) (Navarrete et al. 2016) proposed the use of escalating doses of benznidazole during the first days of treatment to increase its tolerability. However, Galvan et al. (2019) (Galván et al. 2019) presented a study where it cannot be asserted that a strategy of progressive doses is better than the use of full doses from the beginning.

### Visceral involvement

Cardiac involvement was diagnosed if (a) a single electrocardiogram (ECG) or Holter study showed: bradycardia (<50 beats per minute), second-degree atrioventricular block, complete atrioventricular block, another alteration of electrical conduction, or tachyarrhythmia; (b) an echocardiogram showed: left ventricular dysfunction with ejection fraction less than 60%, increased left ventricular diastolic diameter, hypokinesia or akinesia of the ventricular wall, or apical aneurysm; or (c) a cardiac nuclear magnetic resonance study with late gadolinium enhancement (Gascón et al. 2008). Patients were classified from a cardiac point of view according to the three groups proposed by Carrasco et al. (Carrasco et al. 1994): phase I, asymptomatic patients with no electrocardiographic or echocardiographic evidence of cardiac involvement; phase II, asymptomatic patients with electrocardiographic or echocardiographic evidence of cardiac involvement; and phase III, patients with heart failure. Gastrointestinal involvement was diagnosed if (a) a barium enema showed a slow transit or contrast retention; or (b) an esophagogram showed esophageal dilatation, megaesophagus, or achalasia. All patients underwent at least one cardiological study with ECG and chest X-ray. The rest of the diagnostic tests were performed according to the patient’s symptoms, ECG results, and medical criteria.

#### RT-PCR procedure

An RT-PCR to detect *T. cruzi* was performed in duplicate before starting treatment in all patients, with DNA in peripheral blood pretreated with guanidine ethylenediaminetetraacetic acid (EDTA). Nucleic acid was extracted using the MagNA Pure Compact Instrument, with the MagNA Pure Compact Nucleic Acid Isolation Kit I (Hoffmann–La Roche, Basilea, Switzerland). For the amplification and detection of *T. cruzi* DNA, the RealStar Chagas PCR Kit 1.0 (Altona Diagnostics GmbH, Mörkenstr, Germany) was used. RT-PCR assays were run on the CFX96 Real-Time PCR Detection System (Bio Rad Laboratories, Hércules, California, USA). The RealStar Chagas PCR Kit 1.0 is a qualitative test for targeting kinetoplast DNA (kDNA), and analytical sensitivity is 2.8 copies/μL eluted [95% confidence interval (CI): 2.5–3.4 copies/μL]. This assay has been internally validated by the manufacturer for in vitro diagnostic use in line with Regulation (EU) 2017/746 as declared by the “Conformité Européenne—in vitro diagnostics” (CE-IVD) label; however, assessment details have not been made publicly available. Previous work obtained a quantification limit in the PCRs with a molecular target is kDNA of 0.9 parasites/mL (Ramírez et al. 2015).

No deviations from the manufacturers’ instructions were introduced with regard to the interpretation of the real-time PCR signals. A negative sample was considered valid when the internal control was amplified with a Ct < 40 and inhibited when the criterion was not fulfilled. A sample was considered positive when the Ct of the target was < 45.

### Assessment of cytokines profile

Serum samples were collected and remained frozen at −80°C until the assay. Concentrations of the following cytokines were evaluated: IFN- γ, IL-12p70, IL-1β, IL-2, IL-4, IL-6, TNF-α, IL-17A, and IL-5, using the High Sensitivity 9-Plex Human ProcartaPlex Panel immunoassay kit (Thermo Fisher Scientific Inc, Waltham, MA, USA). The analysis was performed by Multiplex MAGPIX with Luminex methodology (EMD Millipore, Temeluca, California, USA). Because the cytokine levels were abnormally low, we repeated the procedure 6 months later on the same samples, with similar results. We cannot rule out sample degradation during the process of collection and storage. The period between collection and freezing at −80°C ranged from 2 months to 24 months.

### Statistical analysis

Categorical variables were described as frequencies (absolute value and percentage) and analyzed using the χ^2^ with Yates correction and Fisher’s exact test. For quantitative variables, we assessed the normality of the distribution by means of the Kolmogorov-Smirnov or Shapiro-Wilk test, depending on the number of variables. Non-parametric quantitative variables were described as median and range and compared using the Mann-Whitney *U* test. *P* values of less than 0.05 were considered statistically significant. All analyses were undertaken using IBM SPSS Statistics for Windows (version 25; SPSS Inc, Chicago, IL, USA).

### Ethical aspects

The ethics committee of the Alicante General University Hospital approved the study protocol (CEIC PI2015/15). Written informed consent was obtained from all participants, and all procedures were performed in accordance with the ethical principles reflected in the Declaration of Helsinki.

## Results

Of the 71 patients included in the prospective cohort, 13 were excluded from this study (10 did not have an available sample of serum for the analysis of cytokines, and 3 did not have an available blood sample for doing the RT-PCR). Thus, a total 58 patients with RT-PCR and serum cytokines profiles were analyzed.

### Demographic and clinical characteristics of patients

The median age of participants was 42.5 years (range 22–74); 39 (67.2%) were women, 52 (89.7%) were born in Bolivia, and 29 (50%) had been residing in Spain for more than 15 years. Cardiological symptoms were observed in 19 (32.8%) patients, and cardiac involvement was confirmed in 14 (24.1%) of these: 13 had phase II cardiomyopathy (right bundle branch block [*n*=5], sinus bradycardia [*n*=3], complex ventricular extrasystole [*n*=2], supero-anterior hemiblock [*n*=2], auriculo-ventricular block [*n*=1], left bundle branch block [*n*=1]), and 1 had heart failure (decreases ejection fraction in echocardiogram). Gastrointestinal involvement was detected in one case (1.7%).

Forty-one patients (70.7%) were treated with benznidazole and one (1.7%) with nifurtimox. Seven patients were not treated (four because they did not come to the consult and three because they were older than 55 years of age). Of those treated, the prescribed regimen was completed in 32 (76.2%), suspended due to adverse effects in 8 (19.1%), and abandoned and lost to follow-up in one each (4.8%). Eighteen of the 40 patients treated in follow-up (45.0%) experienced side effects, the most common of which were urticaria/erythema (15%), headache (15%), and abdominal pain (12.5%) (Table 1).

**Table 1:** Sociodemographic, epidemiological, clinical and treatment profile of patients with Chagas disease included in the study (*n* = 58)

Epidemiological and demographic data	n/N (%)
Age, years (median, range)	42.5 (22–74)
Sex, female	39/58 (67.2%)
More than 15 years in Spain	29/58 (50%)
Country of birth	
Bolivia	52/58 (89.7%)
Paraguay	4/58 (6.9%)
Argentina	2/58 (3.4%)
**Questions about Chagas disease**	
Knows about Chagas disease	43/58 (74.1%)
Relatives with Chagas disease	30/58 (51.7%)
Has lived in rural area	36/58 (62.1%)
Transmission mechanism	
Vector-borne	37/58 (63.8%)
Blood transfusion	3 /58 (5.2%)
Unknown	14/58 (25.9%)
Reason for diagnosis	
Screening campaign	14/58 (24.1%)
Routine immigrant screening	7/58 (12.1%)
Pregnancy screening	6/58 (10.3%)
Blood/organ donation	5/58 (8.6%)
**Clinical and treatment**	
Comorbidity*	9/58 (15.5)
Obesity	6/58 (10.3)
Hypertension arterial	2/58 (3.4)
Diabetes mellitus	2/58 (3.4)
Chronic liver disease	2/58 (3.4)
Chronic obstructive pulmonary disease	1/58 (1.7)
Cardiological symptoms *	19/58 (32.8%)
Palpitations	15/58 (25.9)
Chest pain	3/58 (5.1)
Dizziness	2/58 (3.4)
Gastrointestinal symptoms*	21/58 (36.2)
Constipation	15/58 (25.9)
Pyrosis	3/58 (5.1)
Abdominal pain	3/58 (5.1)
Cardiac involvement	14/58 (24.1%)
Gastrointestinal involvement	1/58 (1.7%)
**Treatment and outcome**	
Treated patients	42/58 (72.4%)
Treatment completed	32/42 (76.2%)
Deprescription due to adverse events	8/42 (19.1%)
Loss to follow-up	1/42 (2.4%)
Treatment abandonment	1/42 (2.4%)
Adverse events *	18 /40 (45.0%)
Urticaria	6/40 (15.0)
Headache	6 /40 (15.0%)
Abdominal pain	5/40 (12.5%)
Neuropathy	3/40 (7.5%)
Nausea	3/40 (7.5%)
Arthralgia-myalgia	2/40 (5.0%)
Asthenia	1/26 (2.5%)
Days of treatment (median, range)	
Treatment completed	66 (54–112)
Treatment uncompleted	38 (7–63)

*Sum of all cases is more the total because the same patient can have more than one disease.

RT-PCR in pre-treatment peripheral blood was positive for *T. cruzi* in 17 (29.3%) patients: 13 patients were in the indeterminate phase of the disease (30.2% of those with an indeterminate form), and 4 patients had cardiac involvement (28.6% of those with a cardiac form).

When analyzing the serum cytokine levels, patients with positive *T. cruzi* RT-PCR in peripheral blood had a higher median concentration of TNF-α (*p* = 0.003), IL-6 (*p* = 0.021), IL-4 (*p* = 0.031), IL-1β (*p* = 0.036), and IL-17A (*p* = 0.043) than those with a negative test (Table 2).

**Table 2:** Median (range) serum cytokine concentrations according to *Trypanosoma cruzi* RT-PCR result in patients with Chagas disease

Cytokines (ng/mL)	Positive *T. cruzi* RT-PCR (*N*=17) Median (range)	Negative *T. cruzi* RT-PCR (*N*=41) Median (range)	*P* value
IFN-γ	0.11 (0.10–249)	0.10 (0.10–184)	0.37
IL-12p70	0.09 (0.11–101.0)	0.11 (0.01–450)	0.21
IL-1β	0.09 (0.00–820)	0.12 (0.00–289)	**0.036**
IL-2	0.15 (0.01–966)	0.14 (0.01–929)	0.12
IL-4	0.13 (0.02–343)	0.02 (0.00–100)	**0.031**
IL-6	220 (0.00–2382)	0.28 (0.15–3001)	**0.021**
TNF-α	0.51 (0.01–500)	0.01 (0.00–0.80)	**0.003**
IL-10	0.09 (0.01–185)	0.12 (0.00–118)	0.35
IL-17A	0.17 (0.01–1645)	0.13 (0.00–1249)	**0.043**
IL-5	0.01 (0.00–125)	0.04 (0.00–442)	0.39

*PCR*, polymerase chain reaction. Statistically significant (*p* < 0.05) values in bold.

When comparing serum cytokine concentrations, patients with cardiac involvement (*n*=14) had a higher median concentration of IL-5 (*p* = 0.014) than those without (Table 3). Our cohort included only one case of digestive involvement, so the serum cytokine profile was not studied.

**Table 3:** Median (range) serum cytokine concentrations according to cardiac involvement in patients with Chagas disease

Cytokines (ng/mL)	With cardiac involvement (*n*=14)	Without cardiac involvement (*n* = 44)	*P* value
IFN-γ	0.10 (0.01–184)	0.20 (0.01–249)	0.210
IL-12p70	0.12 (0.01–101)	0.11 (0.01–450)	0.053
IL-1β	0.11 (0.01–820)	0.12 (0.01–890)	0.504
IL-2	0 .13 (0.00–965)	0.11 (0.01–514)	0.648
IL-4	0.09 (0.01–343)	0.08 (0.01–226)	0.383
IL-6	0.13 (0.01–2020)	0.14 (0.00–3001)	0.310
TNF-α	0.04 (0.01–300)	0.03 (0.00–500)	0.691
IL-10	0.02 (0.01–185)	0.03 (0.02–118)	0.191
IL-17A	0.12 (0.02–1645)	0.10 (0.05–500)	0.281
IL-5	0.13 (0.01–442)	0.02 (0.00–141)	**0.014**

Statistically significant (*p* < 0.05) values in bold

## Discussion

This study explored the serum cytokine profile of 58 patients chronically infected with *T. cruzi* and living in a non-endemic country. Patients with detectable parasitemia measured by RT-PCR in peripheral blood had a higher concentration of TNF-α, IL-1β, IL-4, and IL-6, whereas those with negative RT-PCR showed elevated levels of IL-17A.

The pro-inflammatory cytokines (Th1 immune response) (IL-1, IL-2, IL-6, IL-8, IL-18, IFN-γ, and TNF-α) generally regulate growth, cell activation, differentiation, and homing of the immune cells to the infection sites. Anti-inflammatory cytokines (as IL-4, IL-10, IL-12, IL-17, IL-22, IL-37, IL-38) (Th2 immune response) are instrumental for controlling inflammatory cytokine production (Turner et al. 2014). A distinct profile of cytokines and chemokines has been described in patients with Chagas disease. In animal models, Th1 immune response is crucial in the acute phase of Chagas disease to control the parasitemia (Teixeira et al. 2002). The Th2 immune response is relevant for modulating the disease in indeterminate Chagas (Souza et al. 2004).

Few studies have investigated the cytokines and chemokines profile in serum or plasma in patients with and without *T. cruzi* parasitemia. Volta et al. (Volta et al. 2016) analyzed 35 infants with congenital *T. cruzi* infection: 15 were diagnosed by detection of parasites by microscopy in the first month after delivery; 10 in the sixth, and 10 by the presence of *T. cruzi*-specific antibodies at 10 to 12 months. Infants who did not develop congenital *T. cruzi* infection had higher levels of IFN-γ than infected infants born to uninfected mothers. IFN-γ and monocyte chemoattractant protein-1 (MCP-1) production induced in *T. cruzi*-infected infants correlated with parasitemia, whereas the plasma levels of IL-17A, IL-17F, and IL-6 were less parasite load-dependent.

Several years later, Volta’s research group (Volta et al. 2021) studied 30 *T. cruzi-*infected nonpregnant women, 35 mothers of noninfected children, and 35 infected mothers of infected babies. In *T. cruzi-*infected nonpregnant women, IL-12p70 IL-15, IFN-γ, and TNF-α levels were positively correlated with parasitemia, whereas IL-10 was not. In mothers of noninfected children, parasitemia showed a positive correlation with the levels of IL-12p70, IL-15, IFN-γ, and TNF-α and a negative correlation with IL-6. In mothers of babies congenitally infected with *T. cruzi*, parasitemia was positively correlated only with IL-15 and inversely correlated with IL-1β.

Llaguno et al. (Llaguno et al. 2011) studied 29 chronic Chagas patients with parasitemia (17 cases of indeterminate Chagas and 12 cases of Chagas cardiomyopathy). They observed that Chagas cardiomyopathy was associated with higher median values of IL-10 and lower levels of IFN-γ compared to indeterminate Chagas.

Salvador et al. (Salvador et al. 2020) studied 45 patients with Chagas disease (19 with parasitemia and 26 with-non parasitemia), observing that patients with parasitemia had a higher median concentration of IL-10 and IL-1β and a lower median concentration of IL-8.

In our study, there was a higher concentration of the pro-inflammatory cytokine TNF-α, IL-1β, IL-6, and IL-4 (cytokines inducing differentiation of naive helper T cells) in patients with positive RT-PCR results for *T. cruzi*. Our results are similar to Salvador et al.’s (Salvador et al. 2020) and Volta et al.’s (Volta et al. 2021), where IL-1β and TNF-α were higher in women with parasitemia. IL-6 was higher in patients with parasitemia, but in Volta et al.’s (Volta et al. 2016) study in newborns, those with less parasite load had higher IL-6 plasma levels. In murine models, IL-6 mediates antiparasitic protective responses against *T. cruzi* (Llaguno et al. 2011). Also in our study, those with negative RT-PCR showed elevated levels of IL-17A, which is consistent with Volta et al.’s (Volta et al. 2016) results in newborn, where the plasma levels of IL-17A were associated with less parasite load.

A recent case-control study and meta-analysis identified an IL-10 polymorphism, the genotype TT at -819 rs1800871, as a contributor to genetic susceptibility in chronic cardiac cardiomyopathy, making this polymorphism a suitable candidate to be included in a panel of predictive biomarkers of disease progression (Grijalva et al. 2022). These genetic variations in the IL-10 gene promoter induce high IL-10 production (Grijalva et al. 2022).

Different studies in endemic countries have reported variable results regarding the potential association between plasma or serum cytokines expression and Chagas cardiomyopathy. This heterogeneity is due to differences in criteria for Chagas cardiomyopathy, its severity, the procedures for measuring cytokines (e.g. ELISA, flow cytometry assay), the type of cytokines analyzed (pro-inflammatory, anti-inflammatory), and the comparison (or not) with indeterminate cases, among others.

Gomes et al. (Gomes et al. 2003) studied 70 patients with Chagas cardiomyopathy and 40 with indeterminate Chagas, analyzing the expression of cytokines (in vivo) through peripheral blood mononuclear cell culture. They observed the production of IFN-γ by CD3^+^ CD4^+^ cells in cardiomyopathy, along with the production of IL-10 by macrophages/monocytes, leading to regulation of the immune response, in indeterminate patients (Gomes et al. 2003). In López et al.’s (López et al. 2006) study of 64 patients with Chagas disease, the patients with severe (phase III) Chagas cardiomyopathy had higher levels of IL-6 than patients in phase I (indeterminate). Vasconcelos et al. (Vasconcelos et al. 2015) analyzed 35 patients with mild-moderate Chagas cardiomyopathy, 26 patients with severe Chagas cardiomyopathy, and 18 patients with indeterminate Chagas, finding that serum IL-10 levels in patients with Chagas cardiomyopathy were higher than those in indeterminate patients, especially in those with mild-moderate Chagas cardiomyopathy

On the other hand, Sousa et al. (Sousa et al. 2014) studied cytokine levels in 82 patients with indeterminate Chagas and 94 with dilated Chagas cardiomyopathy. The individuals with an indeterminate state had higher IL-10 expression, whereas the Chagas cardiomyopathy group presented the highest inflammatory cytokine expression (IFN-γ, TNF-α, IL-6 and IL-1β). More recently, Wang et al. (Wang et al. 2021) measured multiple pro-inflammatory cytokines in 94 patients with Chagas myocardiopathy, 48 patients with dilated cardiomyopathy from idiopathic causes, and 25 controls. Investigators found significantly higher stem cell growth factor beta (SCGF beta), hepatocyte growth factor (HGF), monokine induced by interferon gamma (CXCL9), and macrophage inhibitory factor (MIF) in Chagas myocardiopathy patients with advanced heart failure compared to the control group.

Cytokines levels may change with treatment. De Sousa et al. (de Sousa et al. 2019) studied 40 people affected by chronic Chagas myocardiopathy, finding lower serum IFN-γ concentrations in patients receiving angiotensin-converting enzyme inhibitors. Patients using amiodarone and aldosterone antagonist presented higher serum TNF-α concentrations. However, levels of IL-10 and IL-17 did not differ according to treatment.

Bestetti et al. (Bestetti et al. 2019) studied 13 patients with Chagas myocardiopathy, 22 patients with Chagas myocardiopathy and arterial hypertension, and 28 controls. They found that patients with Chagas myocardiopathy and arterial hypertension had higher plasma levels of diverse pro-inflammatory, anti-inflammatory, and regulatory cytokines compared to patients with Chagas myocardiopathy alone. The authors concluded that patients with Chagas myocardiopathy and arterial hypertension presented a higher level of immunomodulation.

Poveda et al. (Poveda et al. 2014) studied 16 patients with Chagas cardiomyopathy, 38 non-cardiac chagasic patients and 9 non-infected individuals. The non-cardiac chagasic patients showed higher frequency of IL-13, IL-5, and IL-10 (anti-inflammatory cytokines) and lower frequency of IL-2, IL-6, IL-9 and IL12 (pro-inflammatory cytokines), while the patients with Chagas cardiomyopathy displayed higher pro-inflammatory and lower anti-inflammatory cytokines.

Rodrigues et al. (Rodrigues et al. 2022) studied 50 patients with Chagas myocardiopathy; those with a left ventricular fraction less (LVEF) than 35% had higher IL-8 and IL-12 serum levels, while elevated IL-6 was related to the presence of right ventricular dilation on the echocardiogram. However, in the multivariate model, only IL-1β was a significant predictor of death in those patients.

Other researchers have studied other cytokines, including TNF, sTNFR1 and sTNFR2. Silva et al. (Silva et al. 2020) evaluated 64 patients with Chagas myocardiopathy, reporting that higher plasma levels of TNFR1 and sTNFR2 were associated with worse systolic function.

In our study, patients with Chagas cardiomyopathy presented a higher concentration of the cytokine IL-5, which stimulates B cell growth, increases immunoglobulin IgA, and has long been causally associated with several allergic diseases.

Moreover, there are several studies that show that transforming growth factor beta (TGF-β) modulates the host immune response and inflammation, increases heart fibrosis, stimulates remodeling, and slows heart conduction via gap junction modulation. Pre-clinical studies have shown that TGF-β signaling inhibitors may revert these effects, opening the door to a promising therapeutic approach (Ferreira et al. 2022).

The thickness of epicardial adipose tissue in people with heart disease is also worth considering, since this has been associated with a pro-inflammatory profile (Parisi et al. 2019). Some studies have also reported high IL-6 levels in patients with chronic Chagas cardiomyopathy. A study in Argentina showed that patients with Chagas cardiomyopathy showed major metabolic and hormonal abnormalities, in parallel with increased IL-6 and leptin serum levels (González et al. 2018).

Regarding digestive Chagas (megacolon o megaesophagus), there is scant information about the potential association between plasma or serum cytokines expression and digestive Chagas. In 1999, Ward et al. (Ward et al. 1999) analyzed IL-2, IFN-g, and TNF in 91 patients with different clinical forms of Chagas’ disease, with some groups containing only 4 cases. They did not observe differences in the level of cytokines. Some years later, Pisseti et al. (Pissetti et al. 2009) studied 20 patients with digestive Chagas, myocardiopathy Chagas, indeterminate Chagas, and a control group, observing higher levels of IL-10 in patients with the digestive form. Cardoso et al. (Cardoso et al. 2006) reported that cells from patients with digestive Chagas disease have a distinct cytokine profile compared to patients with other clinical forms of Chagas disease; the authors suggested that eosinophils are the main source of cytokine production in this clinical entity (Sunnemark et al. 1996). Our cohort included only one case of digestive involvement, so the serum cytokine profile was not studied.

The main strength of this study is that it provides pivotal information about the serum cytokine profile and its relationship with parasitemia in peripheral blood, and it was performed in patients with Chagas disease outside endemic areas (non-endemic region). On the other hand, there were few participants, limiting the generalizability of the results, especially with regard to the evaluation of the cytokine profile according to visceral involvement, because most of the patients in the symptomatic chronic phase of the disease had a very incipient presentation. Secondly, and like other European studies, the patients were mainly women of Bolivian origin, making it difficult to extrapolate the results to other Latin American populations and to men. Thirdly, the RT-PCR for *T. cruzi* in peripheral blood was performed only once, so results should be interpreted with caution. Another limitation is that cytokine production in plasma was abnormally low, and its measurement may have been influenced by the type of procedure used for analysis. However, we repeated the procedure and obtained similar results. Finally, we did not compare results with freshly collected samples to ensure that cytokines were not degraded during the collection and storage process. Furthermore, in blood, cytokines production is intermittent, and cytokine-mediated organ damage depends on the cytokines detected in the tissue of patients with Chagas disease. Another consideration is the comorbidity profile, as several diseases present in our study sample can alter the cytokines profile, such as obesity (n=6), COPD (*n*=1), chronic liver disease (*n*=2), and diabetes (*n*=2). These comorbidities may have an impact on the results when comparing the cytokines in Chagas cardiomyopathy. Moreover, this study did not have a control group, which would be of interest to reinforce the strength of the conclusions (Poveda et al. 2014).

Another area of research in the immunology of *T. cruzi* infection is the study of the gene polymorphism of chemokines and chemokine receptors and the association with the development of Chagas cardiomyopathy and digestive pathologies (Flórez et al. 2011, 2012). In addition, it is unclear whether the immunological profile of chronic Chagas disease is modified by living in a non-endemic region. Further studies are needed to confirm and deepen knowledge on immunoregulatory control of the disease in people living in a non-endemic region.

## Conclusions

In our cohort of chronically infected Chagas disease patients, those with detectable *T. cruzi* parasitemia measured through RT-PCR in peripheral blood had a higher concentration of pro-inflammatory cytokines (TNF-α, IL-1β, IL-6 and IL-17A) and IL-4 than those with negative RT-PCR. These results reinforce the key role that cytokines play in parasitemia control.

## Data Availability

J.M. Ramos-Rincon has full access to and is the guarantor for the data. The datasets generated are available from the corresponding author on reasonable request.
